# Survivin expression pattern in the intestine of normoxic and ischemic rats

**DOI:** 10.1186/s12876-017-0625-6

**Published:** 2017-06-14

**Authors:** Alexandra Scheer, Shirley K. Knauer, Rabea Verhaegh

**Affiliations:** 1 Institute of Physiological Chemistry, University Hospital Essen, Hufelandstraße 55, D-45147 Essen, Germany; 2 Institute for Molecular Biology, Centre for Medical Biotechnology (ZMB), University of Duisburg-Essen, Essen, Germany

**Keywords:** Survivin expression, Ischemia, Reperfusion, Injury, Intestine, Immunohistochemistry, Rat

## Abstract

**Background:**

Survivin, a member of the inhibitor of apoptosis protein (IAP) family, regulates mitosis and chromosome segregation. The expression of survivin proceeds during embryonic development and in addition has already been demonstrated in cancer cells. However, there is also evidence of survivin expression in differentiated tissues, including the gastro-intestinal tract of adult rats. A study with human colon specimens exhibited survivin in most basal crypt epithelial cells of normal mucosa. There is rather limited information on survivin expression in the small intestine. In order to paint a more detailed and thus complete picture of survivin expression patterns in the gastrointestinal tract, we used an immunohistochemical approach in normal adult rat small intestinal and ascending colonic tissue. Moreover, to get deeper insights in the regulation of survivin expression after tissue damage, we also studied its expression in mesenteric ischemia-reperfusion (I/R) injury.

**Methods:**

Mesenteric ischemia-reperfusion injury was induced in male Wistar rats (six animals/group) by occlusion of the superior mesenteric artery for 90 min and subsequent reperfusion for 120 min. Paraffin sections of untreated or ischemically treated tissue were assessed immunohistochemically by survivin and Ki-67 staining.

**Results:**

Survivin could be detected in the small intestine and ascending colon of the normoxia group. It was expressed mainly in the epithelial cells of the crypts and only marginally in the villi. The individual small intestinal segments studied revealed comparable staining intensities. Likewise, expression of survivin was detected in the ischemically damaged small intestine and ascending colon. The expression pattern corresponded to the normoxic animals, as far as verifiable due to the existing tissue damage. Comparison of the expression pattern of Ki-67, a protein that acts as a cellular marker for proliferation, and survivin demonstrated a coincidental localization of the two proteins in the small intestinal and ascending colonic tissue.

**Conclusions:**

Survivin was expressed strongly in epithelial cells of small intestinal as well as ascending colonic tissue. Its expression was located in cells with a high proliferation rate and regenerative capacity. This further supports the decisive role of survivin in cell division. Surprisingly, the ischemically damaged small intestinal and ascending colonic tissue showed a comparably high expression level. These results suggest that there is already a maximal survivin expression under normal conditions. However, the intestine is able to maintain the regenerative capacity even in spite of an ischemic injury. These findings reflect the important relevance of an intact intestinal barrier.

## Background

Survivin is an intracellular protein belonging to the family of inhibitors of apoptosis proteins (IAP) [1]. It is the smallest member of the IAP family (molecular weight 16.7 kDa) and in addition to its anti-apoptotic function acts as a pro-mitotic regulator [2, 3]. During mitosis, survivin is localized to different components of the mitotic apparatus [4] and is able to interact with tubulin of the mitotic spindles e.g. in the G2/M phase [5]. Survivin is a pivotal member of the chromosomal passenger complex and as such regulates chromosomal alignment and cytokinesis during mitosis [6, 7]. Due to its dual function as an apoptosis inhibitor and a mitotic regulator, various studies demonstrate an expression of survivin during embryogenesis as well as in cancer cells [1, 8, 9].

More recent evidence suggests that survivin may also be expressed by normally differentiated cells/tissues [10, 11]. Iskandar et al. have already shown a positive immunostaining for the adrenal gland, liver, stomach, small intestine, colon and kidney of the fetal rat [1]. Besides, in human respectively mice fetal tissue survivin is also expressed in lung, heart, endocrine pancreas and thymic medulla [12]. In human normal adult tissue, survivin was first detected in thymus and placenta [13]. Subsequently, it was also detected in the adult liver of mice and in human gastric mucosa [14, 15]. Iskandar et al. described a positive immunostaining for survivin in the kidney and ovary of adult rats. The very weak expression of survivin detected in the small intestine and colon is mentioned only as a side note without detailed information. There are additional studies that have examined the expression of survivin in the colon in more detail [13, 16–18], unfortunately with inconsistent results. While Ambrosini et al. and Kawasaki et al. reported no expression of survivin with regard to human colon, Gianani et al. and de Souza et al. found survivin to be expressed in crypt epithelial cells of human colonic mucosa.

The intestinal epithelium covering the gastrointestinal tract serves as a protective barrier. A disruption of the mucosal barrier results in penetration of bacteria and other toxins from the intestinal lumen into the lymphatic system, the circulatory system and the abdominal cavity. This breakdown of the intestinal barrier could be the gateway to systemic infection, ultimately leading to septic shock [19, 20]. To maintain their structural function and, as a result of this, the mucosal barrier, the small intestine and the colon have a high ability of cell renewal. For this reason one could indeed expect an especially increased expression of survivin in the intestine, in contrast to the report mentioned above. Specifying the tissue localization of survivin, e.g., either within the individual sections of the small intestine and colon or within the individual cell layers/cell types, could additionally be instructive with respect to cell renewal and proliferation. The latter is pivotal not only for healthy tissue homeostasis and barrier maintenance but likewise for regeneration of cell damage. Therefore, we here studied the pattern and intensity of survivin expression in the small intestine and ascending colon of adult rats under normal conditions and under conditions of ischemia-reperfusion injury (I/R). In order to confirm the role of survivin as a regeneration marker, we compared the expression pattern of survivin and the expression pattern of Ki-67, a protein which was detected in 1984 by Gerdes et al. due to its ability of being expressed only in proliferating cells [21].

## Methods

### Chemicals/materials

Hydrogen peroxide, and o-dianisidine were obtained from Sigma-Aldrich (St. Louis, MO) and formalin solution (10%, buffered) and hematoxylin 51260 from Fluka/Sigma-Aldrich (Steinheim, Germany). Isoflurane (Florene) was from Abbott (Wiesbaden, Germany), ketamine 10% from Ceva (Düsseldorf, Germany), lidocaine (Xylocain 1%) from AstraZeneca (Wedel, Germany), and Ringer’s solution Macoflex N from MacoPharma International (Langen, Germany). Portex catheters (0.58 mm inner diameter, 0.96 mm outer diameter) were supplied by Smiths Medical International (Hythe, U.K.). Paraffin was purchased from (Paraplast Tissue Embedding Medium REF 501006) McCormick Scientific (St. Louis, MO), and medical oxygen from Air Liquide (Düsseldorf, Germany).

### Animals

Male Wistar rats (400–500 g) were obtained from the central animal unit of the Essen University Hospital. Animals were kept under standardized conditions of temperature (22 ± 1 °C), humidity (55 ± 5%) and a 12 h-12 h light–dark cycle with free access to water and food (ssniff-Spezialdiäten, Soest, Germany). All animals received humane care according the standards of the Federation of European Laboratory Animal Science Association (FELASA). The experimental protocol was approved by the local committee based on the local animal protection act.

### Anesthesia, analgesia, and surgical procedure

Rats were anesthetized with isoflurane (2% in 100% medical O_2_ at 4 L/min for induction of anesthesia and 1.5–2% in 100% medical O_2_ at 1 L/min throughout the experiment) through face masks connected to a vaporizer (Isoflurane Vet. med. Vapor, Dräger, Lübeck, Germany) and received ketamine (50 mg/kg, s.c.) into the right chest wall for analgesia. Animals remained anesthetized during the whole experiment until they were sacrificed by cardiac incision. After local xylocaine application (5 mg/kg, s.c.), a Portex catheter was placed within the right femoral artery and the right femoral vein. Thereafter, a median abdominal laparotomy was performed and the superior mesenteric artery was occluded for 90 min using an atraumatic mini-bulldog (Aesculap, Tuttlingen, Germany). The ischemic period ended with the removal of the microvascular clamp and reperfusion started (120 min). The complete small intestine and the ascending colon were resected, and at the end of the experiment, animals were sacrificed by cardiac incision under deep isoflurane anesthesia.

### Study groups

The study was performed with six rats per group in a blinded fashion. In the ischemia group the superior mesenteric artery was occluded for 90 min. The normoxia group underwent all surgical procedures but no mesenteric I/R was induced. All animals received 0.9% NaCl solution (5 mL/kg x h) infused with a syringe pump (Perfusor-Secura FT; B. Braun, Melsungen, Germany) during the experimental period to compensate for fluid loss. The following experimental groups were compared (*n* = 6):

- normoxia group (no I/R)
- ischemia group (90 min ischemia/120 min reperfusion)

### Biomonitoring

The systolic and diastolic blood pressure and the heart rate were continuously recorded via the femoral artery catheter that was connected with a pressure transducer and displayed on a monitor. An infusion bag containing Ringer’s solution delivered 3 mL/h to keep the catheter functional. At a systolic pressure below 90 mmHg for more than 5 min, bolus injections of 0.5 mL 0.9% NaCl solution were repetitively administered through the femoral artery catheter up to a maximum volume of 5 mL/kg x h. Heart rates were determined from systolic blood pressure spikes. The core body temperature was continuously monitored using a rectal sensor and was maintained at 37 ± 1 °C by an underlying thermostated operating table and by covering the animals with aluminum foil. Oxygen saturation was recorded continuously using a pulse oximeter placed at the right hind limb. The breathing rate was determined based on the ventilation movements in 10 min-intervals.

### Histopathological scoring of the ischemia-reperfusion injury to the small intestine

For histological examinations, the ascending colon and the complete small intestine were resected and cut into ten segments (I-X) of equal length (10 cm). Cross sections of 1 cm thickness were taken exactly from the middle of the segments and fixed for at least 24 h in formalin (10%, neutral buffered). The sections were embedded in paraffin (Paraplast Tissue Embedding Medium). For preparations and immunohistochemically staining, 1 μm thick sections of the ascending colon and segment III, V (jejunum) and VII (ileum) of the small intestine were prepared on a rotary microtome and mounted on slides. From each slide, ten crypts from the normoxia and the ischemia group were assessed and documented for evaluating the expression of survivin per field in a blinded fashion. Expression intensities in the individual sections of the small intestine and ascending colon within the respective groups were compared.

### Immunohistochemistry and image analysis

Immunohistochemistry was performed on paraformaldehyde-fixed-paraffin-embedded samples according to the manufacturer’s instructions (Novus biologicals, Littleton, USA) as described previously [22, 23]. As primary antibody a polyclonal rabbit anti-Survivin antibody (dilution 1:1000, Novus Biologicals, Littleton, USA) was used. The Ki-67 staining was carried out using a monoclonal anti-Ki-67 cell cycle marker (BioLogo, Kronshagen, Germany) as primary antibody. Visualization of survivin and Ki-67 was performed with the Vectastain ABC Kit (Vector Burlingame, USA) and UltraVision Detection System Large Volume DAB Plus Substrate System (Thermo Scientific, Fremont, USA) according to the manufacturer’s instructions. Hematoxylin was used for counter-staining. Evaluation of immunohistochemistry was performed by light microscopy in a total magnification of 1:400. Survivin expression was determined by using a special Java-based image processing and analysis program (Image J, W. Rasband, National Institutes of Health, USA). Survivin expressing cells were colored brown and considered positive while cells exhibiting a blue staining were counted as negative. The percentage of brown area per crypt was calculated, averaged and statistically evaluated.

### Statistics

Statistical analysis was performed using GraphPad Prism (GraphPad Software, La Jolla, CA, USA). The data are displayed with mean ± SEM. *P*-values < 0.05 were considered significant. To analyze the IHC quantification data, mean values and SEM in both groups as well as for group comparison were computed using the unpaired *t*-test.

## Results

### Small intestine

#### Survivin expression in the normoxia group

**Fig. 1:**
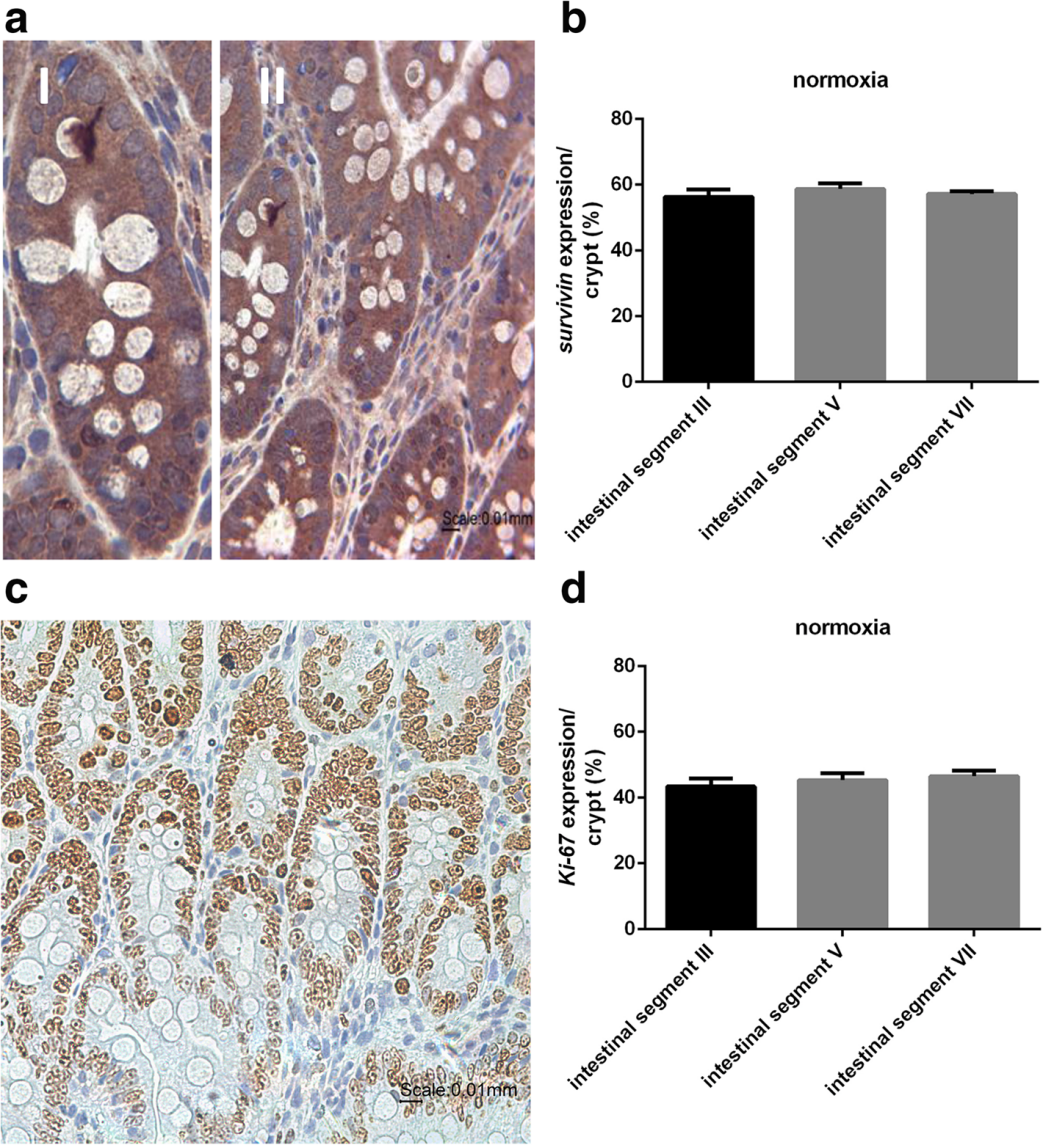
Survivin expression in the normoxia group. **a** Survivin staining of intestinal segment III in the normoxia group (representative figure). Survivin positive cells were immunohistochemically labeled in *brown*. (I): single crypt; (II): small intestine (*jejunum*). Nuclei were stained *blue* by hematoxylin counterstaining. Magnification: 400×. Scale bar: 10 μm. **b** Percentage of survivin expression. The results revealed no significant difference. Values are means ± standard error of the mean (*n* = 6). **c** Ki-67 positive cells were immunohistochemically labeled in *brown*. Nuclei were counterstaining with hematoxylin (*blue*). Magnification: 400×. Scale bar: 10 μm. **d** Percentage of Ki-67 expression. The results revealed no significant difference. Values are means ± standard error of the mean (*n* = 6)

Under normoxic conditions histological examination revealed intact layers of the intestinal wall and all segments of the small intestine showed intact villi and crypts (Fig. 1a). Survivin expression was clearly visible in all intestinal segments studied (III, V (*jejunum*) and VII (*ileum*)). Survivin was specifically expressed in the epithelial cells of the crypts and there was only marginal staining in the villi. The quantitative analysis of survivin expression/crypt revealed a percentage share of 54% in segment III, 59% in segment V and 57% in segment VII (Fig. 1b). Comparison of the expression pattern of Ki-67 and survivin demonstrated a coincidental localization of the two proteins in the small intestinal tissue (Fig. 1c). The percentage of Ki-67 expression was similar in all three segments (segment III 43%, segment V 45% and segment VII 47%) (Fig. 1d).

#### Survivin expression in the ischemia group

**Fig. 2:**
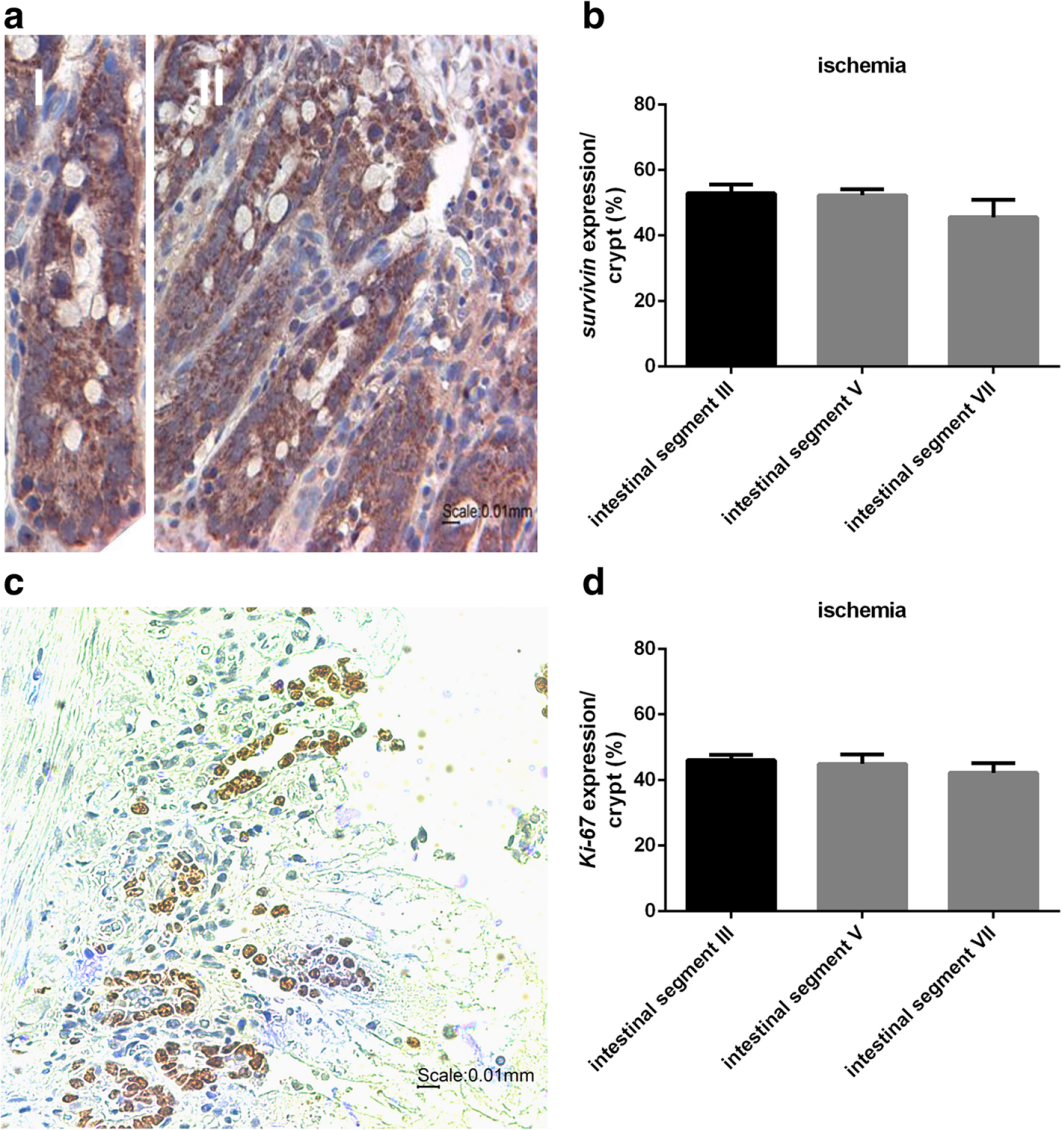
Survivin expression in the ischemia group. a Survivin staining of intestinal segment VII in the ischemia group (representative figure). Survivin positive cells were immunohistochemically labeled in *brown*. (I): single crypt; (II): small intestine (*ileum*). Nuclei were stained blue by counterstaining with hematoxylin. Magnification: 400×. Scale bar: 10 μm (**b**) Percentage of survivin expression. Values are means ± standard error of the mean (*n* = 6). **c** Ki-67 positive cells were immunohistochemically labeled in *brown*. Nuclei were counterstaining with hematoxylin (*blue*). Magnification: 400×. Scale bar: 10 μm. **d** Percentage of survivin expression. Values are means ± standard error of the mean (*n* = 6)

In the ischemic group, the small intestine was severely injured. Microscopic examination of the segments revealed villous destruction, but intact crypt layers were still visible (Fig. 2a). The small intestine injury increased along the jejunum (segment III) until the ileum (segment VII). Survivin was clearly visible in all three intestinal segments studied (III, V *(jejunum)* and VII *(ileum*)). As already demonstrated for the normoxia group, survivin expression was mainly evident in the epithelial cells of the crypts and was only marginally visible in the villi. Segment III showed a percentage share of survivin expression of 56%, segment V of 54% and segment VII of 46% (Fig. 2b). The Ki-67 staining confirmed a similar localization of the Ki-67 and survivin protein as already shown in the normoxia group (Fig. 2c). As in the normoxia group the percentage of Ki-67 expression was similar in all three segments (segment III 46%, segment V 45% and segment VII 42%) (Fig. 2d).

### Colon

#### Survivin expression in the colon of the normoxia and ischemia group

**Fig. 3:**
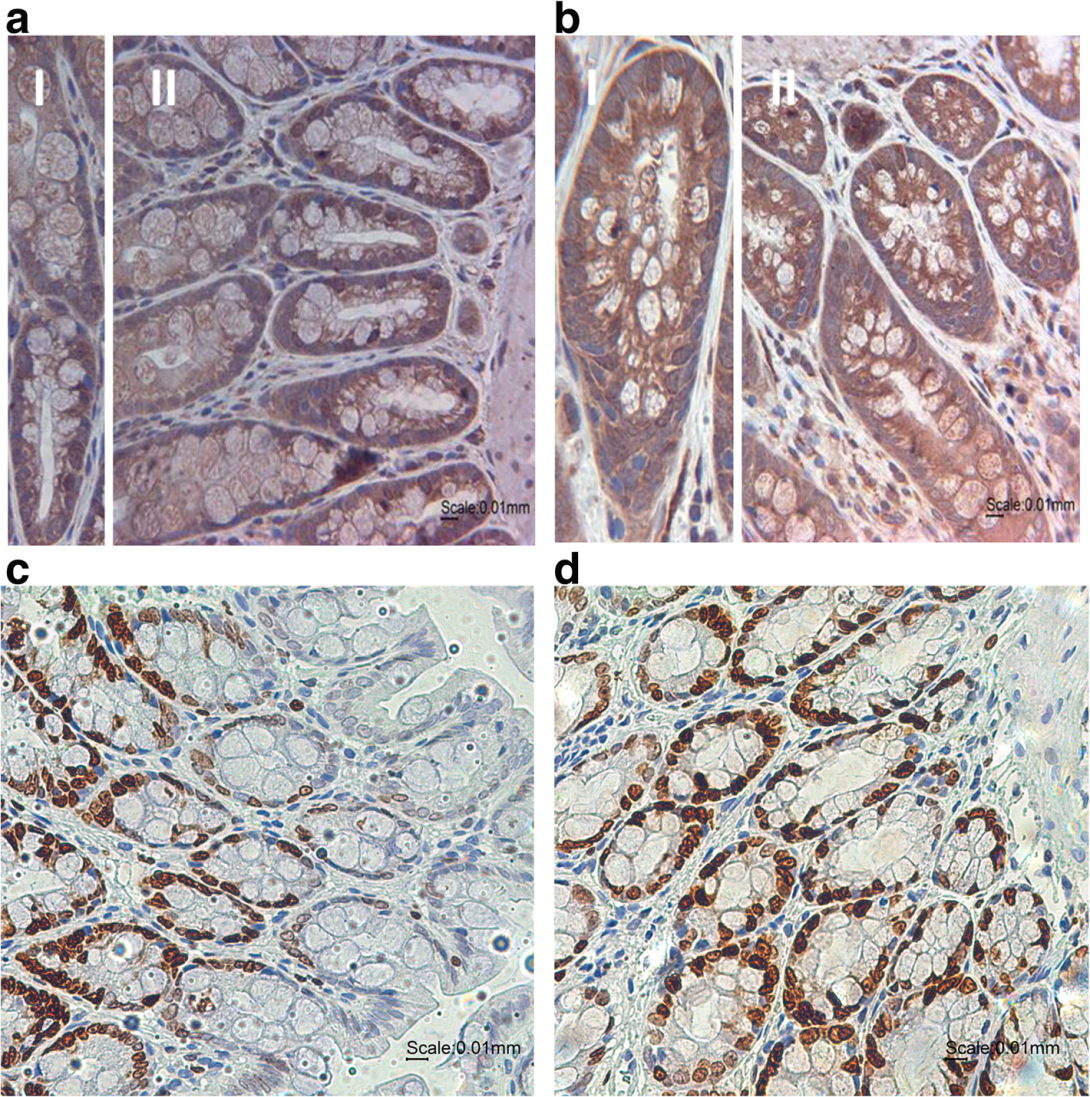
Survivin and Ki-67 staining in the ascending colon. (A/B) Survivin staining of the ascending colon in the normoxia (**a**) and the ischemia (**b**) group (representative figures). Survivin positive cells were immunohistochemically labeled in *brown*. (I): single crypt; (II): ascending colon. Nuclei were counterstaining with hematoxylin (*blue*). Magnification: 400×. Scale bar: 10 μm. (C/D) Ki-67 staining of the ascending colon in the normoxia (**c**) and the ischemia (**d**) group (representative figures). Ki-67 positive cells were immunohistochemically labeled in *brown*. Nuclei were counterstaining with hematoxylin (*blue*). Magnification: 400×. Scale bar: 10 μm

In the normoxic group, the histological examination of the ascending colon revealed almost no injury. The layers of the ascending colon wall and the crypts most widely remained intact. Survivin was specifically visible in the epithelial cells of the crypts (Fig. 3a) and showed a percentage of total survivin expression of 48%. In line with the microscopic examination of the normoxic group, the ischemic group also revealed an almost intact ascending colon. Additionally, survivin expression was also strongly detectable in the ascending colon of the ischemia group. Expression was mainly visible in the crypts (Fig. 3b), with a percentage of 45%. The Ki-67 staining revealed a tissue localization comparable to survivin as already demonstrated for the normoxia and ischemia group of the small intestine (with a percentage of Ki-67 expression/crypt of 34% and 30%, respectively) (Fig. 3c, D).

## Discussion

The epithelial monolayer of the intestinal wall exhibits various physiological functions, such as diffusion exchange and absorption of small molecules. It also acts as a physical, chemical and immunological barrier. To maintain these functions, the epithelial lining of the small intestine and colon is continuously renewed already under normal conditions. Due to the high cell turnover of 2–3 days in the intestinal tissue, a continuous replacement of epithelial cells through a local pool of stem cells in the unimpaired tissue takes place, substantially including the crypt base cells [24]. Their offspring migrate towards the luminal surface, where they are supplied to the terminal differentiation [25, 26].

The findings presented here demonstrate that survivin is expressed both in the small intestine and in the colon without any regional difference between the individual sections of the intestine (e.g. duodenum, jejunum, etc.) (Fig. 1 and 3a). Expression of survivin, however, is exclusively confined to the cells of the epithelial lining (mainly those located within the crypts), i.e. those cells with by far the highest regenerative capacity of all intestinal cells (see above), which is also reflected by the co-expression of the established proliferation marker Ki-67 within these cells (Fig. 1 and 3c). There was no expression of survivin and Ki-67 detectable in any other cell type of the intestine. Preferred expression of survivin in highly proliferating cells such as embryonic cells and adult stem cells or at least in cells with a high regenerative capacity such as tubular epithelial cells or cardiomyocytes is a well-known phenomenon [10, 27–30]. It has been proposed that survivin acts as a mitotic regulator monitoring the correct distribution of chromosomes in dividing cells [4, 5, 7]. This is indeed well in line with the expression of survivin in the intestinal epithelial cells revealed in this study.

To preserve the intestinal tissue architecture an equilibrated balance between proliferation, differentiation and programmed cell loss by apoptosis is needed. Besides survivin, that acts as an apoptosis inhibitor and a mitotic regulator, there are many other mediators of the molecular control of apoptosis. Perhaps one of the most significant among these is the Bcl-2 protein family which delays or inhibits apoptosis. The Bcl proteins regulate the intrinsic initiation of apoptosis by controlling the membrane potential of the mitochondria and the integrity of the mitochondrial membrane. bcl-2, the prototypical anti-apoptotic member of this family, had been reported to be uniform throughout the crypts of both the small and large intestine [31]. In contrast, Merritt et al. reported that the expression of bcl-2 is not uniform in the intestinal epithelia [32]. They found in both mouse and man, bcl-2 to be expressed maximal in the colonic crypt, but greatly attenuated in the small intestine. The differences in the expression of survivin and bcl2 may be due to the fact that bcl-2 in contrast to survivin has no effect on proliferation [33].

Only very weak expression of survivin in the epithelial cells of the small intestine of adult rats and at best a moderate brush border expression in intestinal epithelial cells but less expression in the crypts of normal mouse small intestine has been reported by Iskandar und Al-Joudi [1] and Milcheva et al. [34], respectively, and with regard to human colon even no expression of survivin has been described [13, 17]. These results appear to be rather unlikely considering the well-documented function of survivin as a mitotic regulator in dividing cells. On the other hand, in line with the present results survivin was found to be expressed in crypt epithelial cells of human colonic mucosa [16, 18]. As we observed in rats, the staining intensity decreased from the crypts to the luminal surface [16].

Ischemic damage to the lining of the small intestine occurs remarkably fast after the onset of hypoperfusion. Studies in rats have revealed changes in the villi already three to five minutes after the ischemic injury [24]. The lining of the colon, in contrast, is less easily damaged by a decrease in blood supply and its recovery occurs rather slowly compared to the intestine [35]. In both tissues, however, regeneration after an ischemic insult proceeds by the mechanism of cell renewal/replacement as described above. Here, we observed a severe injury of the small intestinal tissue characterized by loss of villi. However, the crypts remained largely intact. In contrast to the small intestine, the ascending colonic tissue was almost unimpaired. Surprisingly, the level of survivin expression in the ischemically injured small intestine but also in the ascending colon was comparable to normoxic conditions and thus uninjured tissue (for reproducibility only the crypts were compared) (Fig. 1a, 2a and 3a). This observation is indeed remarkable for two reasons. First, it indicates that the regenerative capacity of the intestine remains intact despite of the fact that at least in the small intestine large parts of the tissue are destroyed. It is well known that tissue-specific reduction of survivin impairs the proliferation and maturation of a great variety of cells including hematopoietic cells, neuronal cells, cardiomyocytes, and pancreatic beta-cells [36–39]. Second, it suggests that the expression of survivin and thus the capacity for regeneration of the intestine is already maximal under normal conditions. This is in contrast to observations in several other tissues such as cerebral, hepatic and myocardial tissue where survivin is up-regulated under ischemic/hypoxic conditions [10, 27–29, 40–43]. Furthermore, in different pathological conditions survivin expression is increased, e.g., overexpression of survivin was closely related to tumorigenesis and progression, and was one of the strongest apoptotic inhibitors identified. Survivin is overexpressed in the majority of human cancers, including that of lung, colon, uterus, brain, and ovary, as compared with the normal counterpart [44]. Additionally, it was shown in the inner ear that the severity of the injury is related to the expression of survivin [22, 45]. Temporary hearing impairment caused by moderate noise exposure led to an increase in survivin expression, whereas permanent hearing loss correlates with reduced expression.

Hence, as long as the crypts are intact, which was the case in our study, even in the small intestine regeneration can take place. Like the intestinal epithelium, renal tubular epithelium can completely recover after an ischemic insult. Likewise, an up-regulation of survivin expression could not be detected [10, 46], which is in accordance with our results.

## Conclusions

In summary, survivin was strongly expressed in epithelial cells of small intestinal and ascending colonic tissue. Its tissue expression was restricted to cells with a high proliferation rate and regenerative capacity. This further supports the decisive role of survivin in cell division. The results obtained under ischemic conditions demonstrate a comparably high expression. Hence, the intestine already shows a maximum expression of survivin under normal conditions. Even in case of an ischemia-reperfusion injury, the regenerative capacity of the intestine is maintained. Thereby the intestine is capable of preventing or restoring intestinal barrier dysfunction. These findings emphasize the substantial role of the intestinal barrier and intestinal permeability for health and disease and reflect the important relevance of an intact intestinal barrier.

## Abbreviations

I/R: Ischemia-reperfusion
IAP: Inhibitors of apoptosis proteins
SEM: Standard error of the mean
